# A Rigid–Flexible and Multi-Siloxane Bridge Strategy for Toughening Epoxy Resin with Promising Flame Retardancy, Mechanical, and Dielectric Properties

**DOI:** 10.3390/ijms241814059

**Published:** 2023-09-13

**Authors:** Dingsi Li, Shufeng Lin, Jiahui Hao, Baohan He, Huagui Zhang, Mingfeng Chen

**Affiliations:** Fujian Key Laboratory of Polymer Materials, College of Chemistry and Materials Science, Fujian Normal University, Fuzhou 350007, China

**Keywords:** epoxy resin, intumescent flame retardant, toughening, dielectric property

## Abstract

Developing highly efficient and multifunctional epoxy resins (EPs) that overcome the shortcomings of flammability and brittleness is crucial for pursuing sustainable and safe application but remains a huge challenge. In this paper, a novel biomass-containing intumescent flame retardant containing a rigid–flexible and multi-siloxane bridge structure (DPB) was synthesized using siloxane; 9,10-dihydro-9-oxa-10-phosphaphenanthrene 10-oxide (DOPO); and biomass vanillin. DPB could facilitate the formation of a carbon residual with an intumescent structure, which effectively blocked the propagation of heat and oxygen. As a result, the peak heat release rate (pHRR) and total heat release (THR) of DPB/EP-7.5 decreased by 38.8% and 45.0%, respectively. In terms of mechanical properties, the tensile and flexural elongations at break of DPB/EP-7.5 increased by 77.2% and 105.3%, respectively. Impressively, DPB/EP-7.5 had excellent dielectric properties, with a dielectric constant of 2.5–2.9. This was due to the Si-O bonds (multi-siloxane bridges) contained in DPB/EP, which can quench the polarization behavior of the hydroxyl group. This paper provides a facile strategy for the preparation of multifunctional EP, which will pave the way for the promotion and application of EP in the high-end field.

## 1. Introduction

Epoxy resins (EPs) are used as high-performance polymers with high mechanical strength and transparency as well as superior thermal and chemical stability that are easily processed in a wide range of applications such as automotive manufacturing, aerospace, and circuit encapsulation materials [1,2,3,4]. However, EP has high flammability, and once ignited, the burning speed is very fast. Moreover, the smoke and toxic vapors released by combustion can be extremely harmful to humans, which greatly limits the application range of EP [5,6,7,8].

Generally, halogen-containing additives have often been used to improve the flame-retardant properties in the past few decades, but they have gradually been replaced by halogen-free flame retardants because they would release toxic and harmful halogen-containing gases during combustion and damage the mechanical properties of EP [9,10]. Intumescent flame retardants (IFRs) stand out among halogen-free flame retardants because of their smoke suppression, thermal insulation, and low toxicity [11,12]. An IFR usually consists of three elements: an acid source, a carbon-forming agent, and a foaming source. In the event of a fire, the IFR causes the carbon layer of the substrate to expand, thus protecting the underlying material from the effect of the flame [13]. However, due to the low flame-retardant efficiency of conventional IFR formulations (e.g., the ammonium polyphosphate (APP)–pentaerythritol–melamine system), it is often necessary to add a higher proportion of a typical IFR into the polymer matrix to meet fire safety requirements, which negatively affects the mechanical properties of the material [14]. Shao et al. [15] successfully prepared zeolitic imidazole framework-67 (ZIF-67) by coordinating a reaction among Co^2+^, phosphate, and 2-methylimidazole and dispersed it homogeneously on APP. Compared with pure EP, the pHRR and SPR of 5ZIF-67@APP/EP were reduced by 75.9% and 63.1%, respectively, while the tensile strength and flexural strength were increased by 19.0% and 43.2%, respectively, compared with 5APP/EP. Qin et al. [16] synthesized the new two-dimensional (2D) supermolecule melamine trimetaphosphate (MAP) using melamine (MA) and sodium trimetaphosphate (STMP) as raw materials. The EP-MAP composite required only 4% MAP to achieve an LOI value of 30.0% and a UL-94 V-0 rating with a 65.6% reduction in the peak exothermic rate, while the flexural strength and flexural modulus were increased by 52.7% and 92.2%, respectively. Although many reported studies have improved the mechanical properties while greatly increasing the efficiency of the flame retardant, they still fall short in terms of sustainability. The vast majority of raw materials are non-renewable chemicals, which is incompatible with the concept of sustainable resource development and environmental protection. Recently, biomass materials have been increasingly used in the field of flame-retardant polymers due to their abundance and sustainability [17,18]. There have been many reports focused on biomass materials, including castor oil [19], cellulose [20], and lignin [21], being used as components of IFRs. Cheng et al. [22] used a surface modification of APP with biobased arginine (Arg) to enhance the flame retardancy of EP. The results showed that the EP/Arg-APP composite had a higher LOI value of 34.7%, passed the V-0 rating in UL-94, and reduced the pHRR and TSP by 83.5% and 61.1%, respectively, compared to pure EP, but its tensile strength (down to 32.7 MPa) and elongation at break (down to 32.7%) were significantly reduced. Zhang et al. [23] used a biomass phytate to modify metal–organic framework (MOF) materials and then combined them with β-cyclodextrin to prepare the intumescent flame retardant βCD@P-MOF. The pHRR and SPR of the EP composites were reduced by 41% and 62%, respectively, but their mechanical properties could only remain similar to pure EP and were not effectively improved. Li et al. [24] prepared a highly efficient intumescent flame-retardant EP using green calcium gluconate (CaG) in combination with ammonium polyphosphate. The prepared EP81 passed the UL-94 V-0 rating and achieved an LOI value of 31.8%. The pHRR and TSP were decreased by 39.7% and 49.2%, respectively, compared with EP/10 wt% APP. However, the flexural strength decreased by 29.6%. Although the above works used renewable biomass materials to produce efficient IFRs, the mechanical properties were destroyed to some extent and were not maintained well. Furthermore, with the advent of communication technology and the new energy age, EP as a traditional adhesive and encapsulant material also needs to have the corresponding special properties to keep pace with developments. For instance, a low dielectric constant and low dielectric loss are indispensable properties in the electrical and electronic fields. However, the dielectric constant of traditional epoxy resin is 4–6 [25,26], which cannot be used in the field of high-precision electronics. Na et al. [27] reduced the dielectric constant of epoxy resin by introducing a smaller dipole C-F bond into epoxy resins, which made the molecule harder to polarize. The results showed that the dielectric constants were all significantly reduced (down to 3.38), but they were deficient in terms of flame-retardant performance properties.

Herein, we have designed and synthesized a novel biomass-containing intumescent flame retardant containing a rigid–flexible and multi-siloxane bridge structure (DPB) to fabricate a toughened and strengthened epoxy resin (DPB/EP) with excellent flame retardancy. Synchronously, the prepared DPB/EP achieved impressive results in terms of dielectric properties. In addition, the possible toughened and flame-retardant mechanism was further evaluated using XPS, SEM, and TG-IR.

## 2. Results and Discussion

### 2.1. Characterization of the Structure of DPB

**Figure 1:**
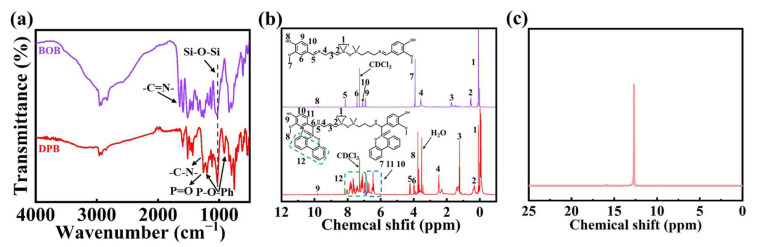
FTIR spectra of (**a**) BOB and DPB. ^1^H NMR spectra of (**b**) BOB and DPB. ^31^P-NMR spectrum of (**c**) DPB.

The structures of BOB and DPB were characterized using FTIR, ^1^H NMR, and ^31^P NMR. Figure 1a shows the FTIR spectra of BOB and DPB, with an absorption peak at 1640 cm^−1^, indicating the presence of C=N bonds in BOB [28,29]. The absorption peak at 1040 cm^−1^ was attributed to Si-O-Si stretching vibrations, and the broad band around 3000 cm^−1^ was attributed to -OH vibrations. Many differences were observed in the FTIR spectra between BOB and DPB. A new characteristic peak (1273 cm^−1^) appeared in the infrared spectrum of DPB, which belonged to -C-N-, and the peak at 1250 cm^−1^ was ascribed to the P=O bond. The peaks at 1190 and 914 cm^−1^ were attributed to the P-O-Ph stretching vibration [30,31]. Figure 1b shows the ^1^H NMR spectra of BOB and DPB, and the peak at 8.14 ppm was attributed to the proton in -CH=N-. The peaks at 9.81 ppm were attributed to the protons in the -OH. The peak occurring at 2.48 ppm was assigned to the proton in -N-CH_2_-, and the peaks at 0.34 ppm were attributed to the protons in -Si-CH_2_-. The peak occurring at 0.08 ppm was attributed to -Si-CH_3_. Compared with BOB, there were new peaks appearing in the ^1^H NMR spectrum of DPB. The peak at 4.24 ppm belonged to the proton in -NH-CH_2_-, which illustrated the additional reaction between P-H and the C=N bond. The peaks at 6.79–8.05 ppm were attributed to the protons of the benzene ring. The ^31^P NMR spectrum of DPB is shown in Figure 1c. There was one peak observed around 12.7 ppm in the ^31^P NMR spectrum of DPB, which further illustrated the successful reaction between BOB and DOPO. Furthermore, the elemental content of DPB (C_50_H_58_O_9_N_2_P_2_Si_2_) (calculated/experimental, wt%) also confirmed the structure of DPB: C, 63.27/63.29; H, 6.16/6.19; O, 15.17/15.20; N, 2.95/2.91.

### 2.2. Thermal Properties of DPB/EP

**Figure 2:**
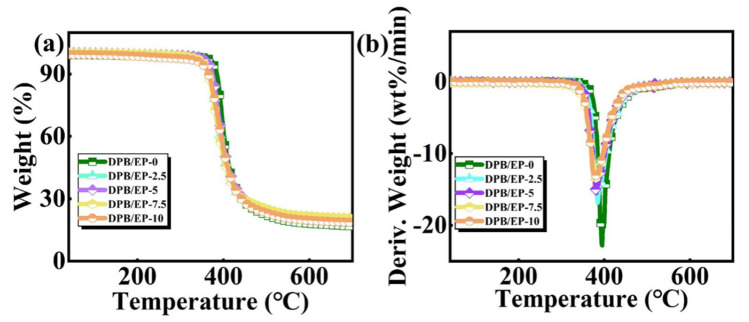
(**a**) TGA and (**b**) DTG curves of DPB/EP.

The thermal stability of DPB/EP was analyzed using a TGA, and the results are shown in Figure 2 and Table 1. It can be observed that DPB/EP showed a trend towards one-step thermal decomposition. (T_5%_ refers to the temperature at 5% weight loss, T_max_ refers to the temperature at the maximum weight loss rate, and RC_700_ refers to the carbon residue rate at 700 °C). From Figure 2a, it can be observed that the T_5%_ of DPB/EP gradually decreased with the addition of DPB, which was due to the poor stability of the P=O bond in DPB, and the catalytic effect of the phosphorus-based acids generated by the decomposition led to the premature degradation of the EP chain [32]. In addition, the RC_700_ of DPB/EP increased slightly with the increase in DPB content, and the char residue of DPB/EP-7.5 was as high as 20.7 ± 0.2% and increased by 3.5% compared with DPB/EP-0 (17.2 ± 0.2%), which indicated that DPB could facilitate the formation of a char layer during the combustion process. This was because the biomass component (vanillin) in DPB acted as a carbon source, while the phosphorus and silicon elements in DPB could play a role in increasing the residual carbon rate of the EP during the combustion process [33,34]. It can be clearly seen from the DTG curves that the maximum weight loss rate of DPB/EP-7.5 decreased from the 22.9 ± 0.7%/min of DPB/EP-0 to 13.5 ± 0.3%/min. This was because the P=O bond in the DPB structure would decompose during combustion to generate phosphorus-containing substances such as phosphorus oxide compounds and phosphoric acid derivatives, which would act as dehydrating agents and promote the formation of a carbon layer [35,36]. Based on the above analysis, DPB increased the carbon residue yield of EP, which blocked heat transfer and improved the safety in the event of a fire.

### 2.3. Combustion Behavior of DPB/EP

**Figure 3:**
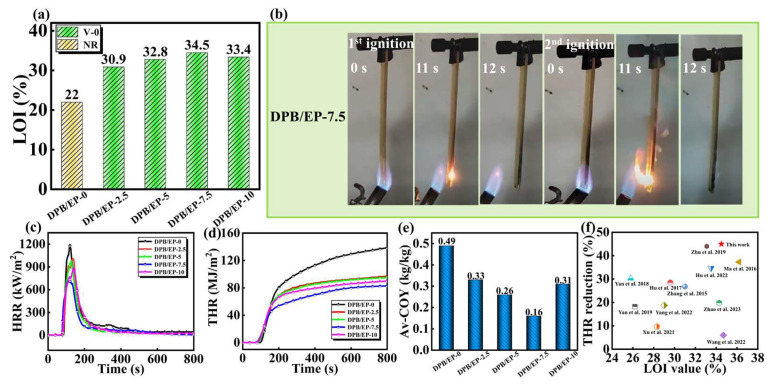
(**a**) LOI and UL-94, (**b**) UL-94 testing screenshot photos from video of DPB/EP-7.5, (**c**) HRR, (**d**) THR, (**e**) av-COY, and (**f**) comparison of the THR reduction values of DPB/EP-7.5 and its corresponding LOI value with previously reported intumescent flame-retardant epoxy resins [39,40,41,42,43,44,45,46,47,48,49].

The flame resistance of DPB/EP was assessed using a vertical burning test and the limiting oxygen index (Figure 3a). By introducing the novel biomass-containing intumescent flame retardant containing a rigid–flexible and multi-siloxane bridge structure (DPB), higher LOI values and UL-94 ratings for the flame-retardant epoxy resin (DPB/EP) were correspondingly obtained. When the content of DPB was just 2.5 phr, the LOI value of DPB/EP-2.5 had a significant improvement compared with DPB/EP-0, from 22% to 30.9%, and the UL-94 already achieved a V-0 rating. Further increasing the content of DPB, the LOI value of DPB/EP-7.5 was enhanced to 34.5%, and the UL-94 had a V-0 rating with excellent self-extinguishing capability, as shown in Figure 3b. The fire behaviors of DPB/EP were further analyzed using a cone calorimeter test (CCT). Corresponding data, including time to ignition (TTI), THR, the mass loss rate (MLR), pHRR, and total smoke production (TSP), are presented in Table 2. Curves of the heat release rate (HRR), THR versus time, and average carbon monoxide yield (av-COY) are shown in Figure 3c–e. The pHRR as well as THR of DPB/EP decreased gradually with the addition of DPB and showed the lowest values in DPB/EP-7.5 (reduced by 38.8% and 45.0%, respectively). The MLR reflects the degree of combustion, and the MLR values of DPB/EP also decreased with the increase in DPB, suggesting that the addition of DPB could improve the combustion degree of EP. In addition, according to statistics, more than 50% of casualties from fire are caused by toxic gases and smoke [32]. The addition of DPB was conducive to reductions in TSP and av-COY, thereby improving the problem of the massive release of toxic gases in the combustion process. The addition of DPB effectively reduced the production of the toxic gas carbon monoxide by 67.3%, which considerably enhanced the chances of safe escape from a fire. The main reason was that substances such as the phosphoric acid derivatives formed by phosphorous compounds during the combustion process could promote dehydration and carbonization. The resulting carbon layer isolated air and heat from the outside world and reduced the release of the flammable gas produced by the thermal decomposition of the polymer. At the same time, the scavenging of hydroxyl radicals by the PO· radical allowed DPB to exert excellent flame-retardant effects in the gas phase as well [37,38]. All the above results showed that lower THR and CO yield values were obtained with the incorporation of DPB, which meant higher fire safety for the DPB/EP thermosets. As shown in Figure 3f, when comparing the THR reduction value of DPB/EP-7.5 and its corresponding LOI value with other studies on intumescent flame-retardant epoxy resins [39,40,41,42,43,44,45,46,47,48,49], it was found that the DPB prepared in this paper showed a more outstanding comprehensive performance of improved LOI values and THR reduction.

### 2.4. Analysis of the Flame-Retardant Mechanism of DPB/EP

#### 2.4.1. Gas Phase Analysis

**Figure 4:**
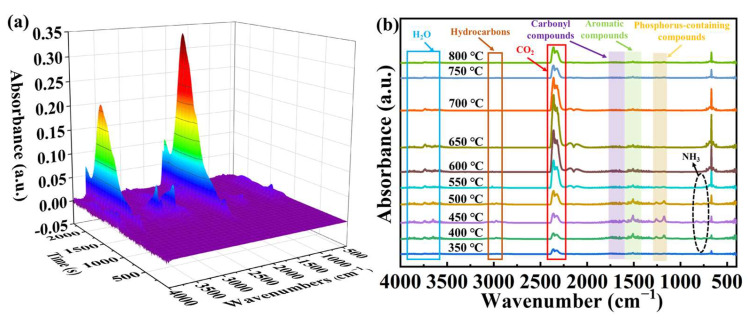
(**a**) Three-dimensional spectra of TG-FTIR for DPB/EP-7.5 and (**b**) FTIR spectra of the pyrolysis products of DPB/EP-7.5 at different temperatures.

In order to explore the flame retardancy characteristics of DPB in the gas phase, the changes in the pyrolysis products of DPB/EP-7.5 were investigated. As presented in Figure 4a,b, the signals at 2990 cm^−1^, 2360 cm^−1^, 1620 cm^−1^, and 1507 cm^−1^ were the characteristic peaks of hydrocarbons, CO_2_, carbonyl compounds, and aromatic compounds, respectively. It is worth noting that at 400 ℃, DPB/EP-7.5 presented two special signal peaks at 1250 cm^−1^ and 1170 cm^−1^, which belonged to P=O and P-O-C, respectively, indicating that DPB would release phosphorus-containing volatiles in the initial combustion process, which could play a role in flame retardance by captured OH or H radicals in the gas phase [50]. The signal peaks at 821 and 747 cm^−1^ corresponded to NH_3_ and were attributed to the decomposition of DPB, which could act as a gas source to dilute the flammable gases during burning and slow down the combustion [46].

#### 2.4.2. Condensed Phase Analysis

**Figure 5:**
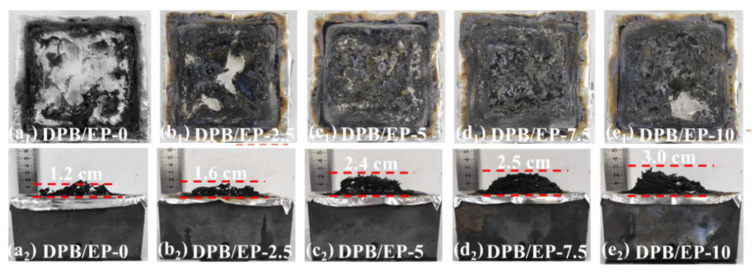
Digital photographs of the char residue of DPB/EP after CCT.

The residual carbon from the cone calorimeter test of the DPB/EP was recorded using digital photographs, as shown in Figure 5. With the addition of DPB, the thickness and integrity of the carbon layer was improved, which could have the effect of insulating and blocking the propagation of combustible gases, thus effectively extinguishing the flame. The phosphorus element in DPB was able to form polyphosphoric acid during combustion to catalyze the formation of carbon [51], while the silicon element was able to form structures such as Si-C and SiO_2_ to enhance the stability of the intumescent carbon layer (shown in XPS). That is to say, the obtained results indicated that DPB was able to promote the formation of intumescent residual carbon.

**Figure 6:**
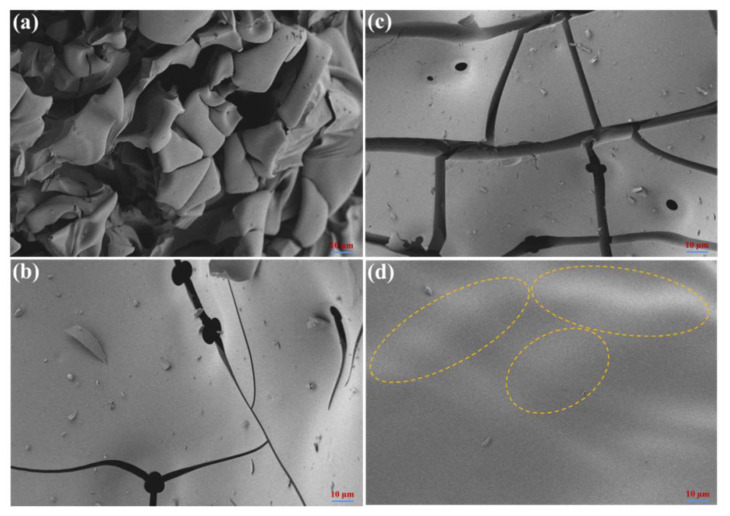
SEM micrographs of char residues for DPB/EP-0 (**a**,**b**) and DPB/EP-7.5 (**c**,**d**) after CCT. Outer surface (**a**,**c**); inner surface (**b**,**d**).

The microstructure of the char layer of DPB/EP after combustion was further analyzed using SEM to determine the flame-retardant mechanism in the condensed phase. As presented in Figure 6a–d, the outer surface of DPB/EP-0 was loose and not compact, while the inner char layer appeared broken, which was not conducive to insulating heat and oxygen. After the addition of DPB, the outer surface was more compact and continuous, and the inner layer had a large number of bulge-like structures, forming a larger expansion structure. The phosphorus element in DOPO supplied an important acid source for DPB to become an expansive flame retardant. The complete and solid carbon layer can prevent the transfer of decomposition products and heat, and further protect the underlying substrate from being ignited by the flame, thus improving the fire safety of EP in practical applications.

**Figure 7:**
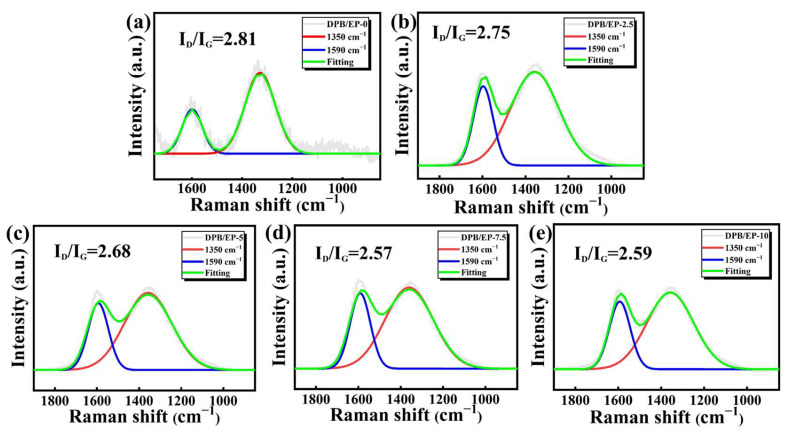
Raman spectra (**a**–**e**) of char residue for DPB/EP.

Figure 7 shows the Raman curves of the char residues, where there were two special absorption peaks at 1350 cm^−1^ and 1590 cm^−1^, belonging to peak D and peak G, respectively. The intensity ratio of the D-band to the G-band (I_D_/I_G_) is usually used to analyze the degree of graphitization of the carbon layer, and a lower ratio of I_D_/I_G_ indicates a higher degree of graphitization and better carbon structures and thermal-oxidative stability [52,53,54,55,56]. The I_D_/I_G_ value of DPB/EP-0 was 2.81, while the values gradually decreased with the addition of DPB, reaching the lowest value of 2.57 when the amount of DPB was 7.5 phr. The above discussion suggests that the addition of DPB was conducive to the formation of more graphitized residual carbon in EP.

**Figure 8:**
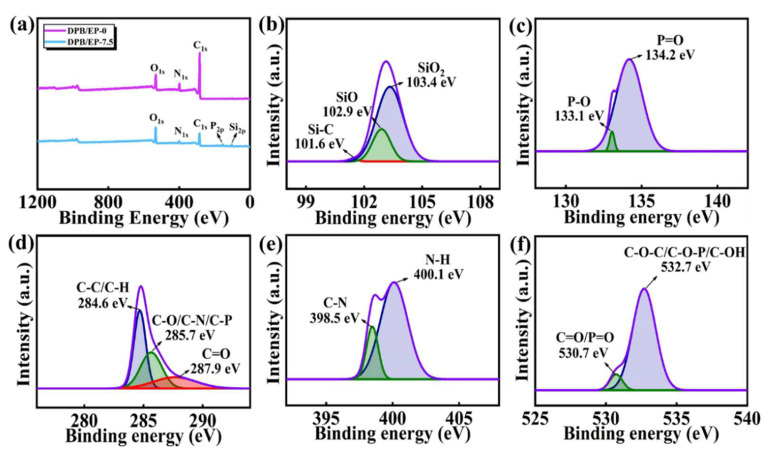
(**a**) XPS spectra of the carbon residues of DPB/EP-0 and DPB/EP-7.5, and the corresponding high-resolution XPS spectra of (**b**) Si_2p_, (**c**) P_2p_, (**d**) C_1s_, (**e**) N_1s_, and (**f**) O_1s_ of the carbon residue of DPB/EP-7.5.

The elemental chemical states of DPB/EP were analyzed using XPS. It can be observed from Figure 8a that DPB/EP-0 contained only C, O, and N, while DPB/EP-7.5 contained P and Si in addition to C, O, and N. Figure 8b–f showed the high-resolution XPS spectra of Si_2p_, P_2p_, C_1s_, N_1s_, and O_1s_, respectively. The spectrum of Si_2p_ was divided into three peaks with binding energies of 101.6, 102.9, and 103.4 eV, corresponding to Si-C, Si-O, and SiO_2_, respectively. The two peaks (Figure 8c) at 133.1 eV and 134.2 eV were deconvolved from the P_2p_ peak and were attributed to P-O and P=O, respectively. The C_1s_ spectrum had three major peaks at 284.6 eV (C-C/C-H), 285.7 eV (C-O/C-N/C-P), and 287.9 eV (C=O). The two intensities of 398.5 and 400.1 eV (Figure 8e) were deconvolved from the N_1s_ peak and were ascribed to C-N and N-H, respectively. Moreover, the deconvolution of the O_1s_ peak in Figure 8f exhibited C=O/P=O (530.7 eV) and C-O-C/C-O-P/C-OH (532.7eV) signals. All of the above results indicated that DPB formed a phospho-oxygen compound and phosphoric acid derivatives during combustion, which contributed to the dehydration and carbonization of EP, forming a protective carbon layer and preventing further combustion.

#### 2.4.3. Flame Retardancy Mechanism

**Scheme 1:**
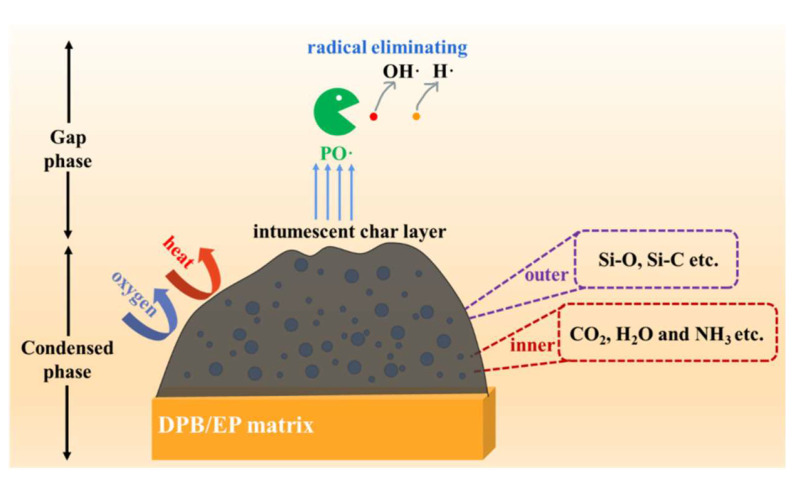
Schematic illustration of the flame-retardant mechanism of DPB/EP.

Based on the above analysis, the flame-retardant mechanism of DPB in EP was proposed (as shown in Scheme 1). DPB exerted an effective flame-retardant effect in both the gas and condensation phases, exhibiting flame suppression and carbon layer barrier effects. In case of fire, the active radicals (P and PO·) generated by DPB would capture H· and OH· radicals when entering the gas phase, thus further terminating the chain reaction of combustion. In addition, the phospho-oxygen compound and phosphoric acid derivatives derived from the thermal decomposition of DPB could effectively promote the dehydration and carbonization of EP. Meanwhile, the non-flammable gases (CO_2_, H_2_O, NH_3_, etc.) released during the decomposition of DPB/EP also promoted the formation of the expanded char layer. And the Si element contained in DPB formed more stable Si-O and SiO_2_, which helped to form a more solid and compact carbon layer, thus isolating the transmission of oxygen and heat. In conclusion, DPB/EP exhibited excellent flame-retardant properties due to the physical barrier, the radical trapping effect of DPB, etc.

### 2.5. Dielectric Properties

**Figure 9:**
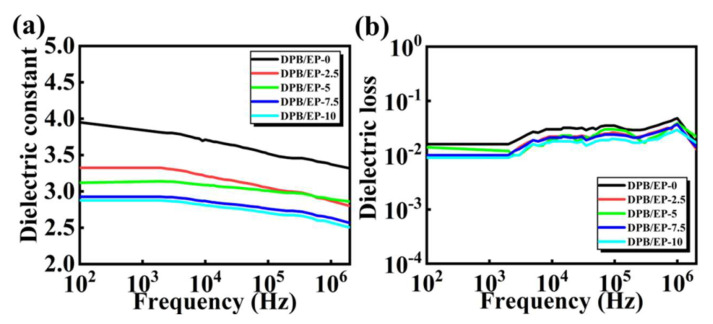
Frequency dependency of (**a**) dielectric constant and (**b**) dielectric loss of DPB/EP in frequency range from 10^2^ Hz to 2 MHz at room temperature.

Nowadays, electronic equipment is used more and more frequently in our daily life, which leads to higher requirements for insulating materials. Among various features, the dielectric constant and dielectric loss are two extremely important parameters for insulating materials. Figure 9a shows the variation in the dielectric constant of DPB/EP with frequency at room temperature. The dielectric constant of DPB/EP decreased significantly with the addition of DPB, with a dielectric constant of 2.5–2.9 when the DPB content was 7.5 phr. This was mainly because the Si-O segment (multi-siloxane bridge) of DPB helped to quench the polarization behavior of the hydroxyl group, which could effectively reduce the dielectric constant of DPB/EP [57,58]. Similarly, the dielectric loss showed a tendency to decrease with the addition of DPB, in line with the results of the dielectric constant, which contributed to a reduction in heat generation in resin-encapsulated electronic devices and improved the safety of the devices.

### 2.6. Mechanical Properties of DPB/EP

**Figure 10:**
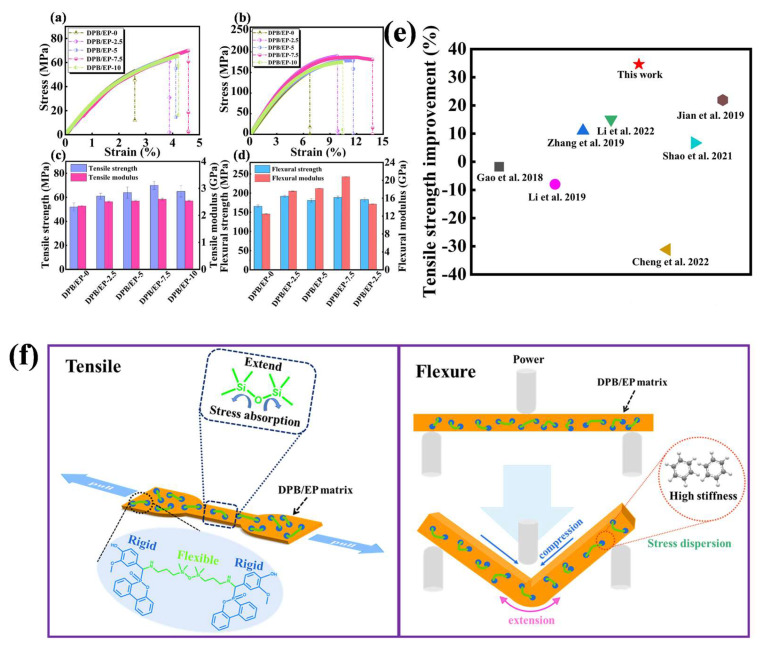
Stress–strain curves of (**a**) tensile and (**b**) flexural tests, (**c**) tensile strength, (**d**) flexural strength, (**e**) comparison of tensile strength improvement of this article and other intumescent flame-retardant epoxy resins in the literature, and (**f**) schematic of the strengthening mechanism of DPB/EP [15,22,24,65,66,67,68].

The rigid and flexible parts contained in the DPB structure are a great help in improving the mechanical properties of EP. It can be observed that the addition of DPB could remarkably improve the tensile strength, flexural strength, and modulus of EP composites. It is noteworthy that the tensile and flexural elongations at break of DPB/EP-7.5 were 77.2% and 105.3% higher than those of DPB/EP-0, respectively (Figure 10a,b), which illustrated that the flexible Si-O bond in DPB played a significant role and could provide a toughening effect for the epoxy resin [59,60,61,62]. Figure 10c,d show the detailed variation in the tensile and flexural values. The tensile strength and modulus of DPB/EP-7.5 reached the maximum values, which were 34.3% and 13.0% higher than those of DPB/EP-0. The flexural modulus shown in Figure 10d also illustrated a similar trend as in Figure 10c. In particular, the flexural modulus of DPB/EP-7.5 was increased by 67.5% compared to DPB/EP-0, which could be attributed to the higher rigidity of DPB. The increases in the strengths and modulus were attributed to the higher aromatic rigidity of DOPO and biomass vanillin in the DPB structure [63,64]. The effects of previously reported intumescent flame retardants on the tensile strength of epoxy resins were compared [15,22,24,65,66,67,68], as shown in Figure 10e. The DPB reported in this paper resulted in a significant increase in tensile strength (34.6%) while it endowed EP with excellent flame-retardant properties. The mechanism by which DPB enhanced the mechanical properties was mainly due to the unique rigid–flexible structure of DPB, which can impart excellent strength as well as toughness to the epoxy matrix. The flexible Si-O bond can absorb some of the energy and stretch when the DPB/EP substrate is subjected to external forces, while the rigid aromatic structure helps to disperse the energy and avoid stress concentration (as shown in Figure 10f).

**Figure 11:**
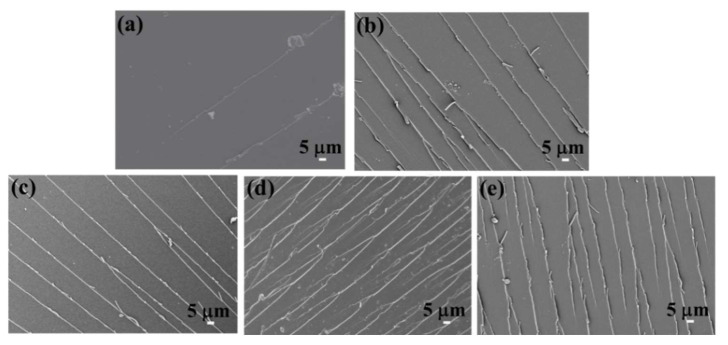
Fracture interfaces of (**a**) DPB/EP-0, (**b**) DPB/EP-2.5, (**c**) DPB/EP-5, (**d**) DPB/EP-7.5, and (**e**) DPB/EP-10 observed using SEM.

It can be observed in Figure 11a that the cross section of DPB/EP-0 was relatively smooth with only a few stripe folds, which was typical for a brittle fracture. However, the number of stripes and folds increased significantly after the addition of DPB, which was of great help to disperse stress and accelerate energy dissipation. The SEM image of the fracture cross section of DPB/EP-7.5 is the most representative and shows an obvious increase in stripes and folds. Such a noticeable change was due to the increased content of flexible Si-O bonds and rigid aromatic rings in the fascinating structure of DPB [63,64], which can effectively absorb and disperse stress, thus overcoming the disadvantage of the brittleness of conventional epoxy resins and providing a practical strategy to further improve the mechanical properties of epoxy resins.

## 3. Methods and Materials

### 3.1. Materials

Vanillin (Van) and anhydrous ethanol (A. R.) were procured from Titan Scientific Co., Ltd. (Shanghai, China). 9,10-dihydro-9-oxa-10-phosphaphenanthrene 10-oxide (DOPO) and 1,3-bis(3-aminopropyl)-1,1,3,3-tetramethyldisiloxane (BTDS) were supplied by Energy Chemical Co., Ltd. (Shanghai, China). Diglycidyl ether of bisphenol A (DGEBA, E-51) was offered by Yunsen Technology Co., Ltd. (Zhangzhou, China). The curing agent of 4,4-diaminodiphenylmethane (DDM); 1,2-dichloroethane ((CH_2_)_2_Cl_2_); and hexane was obtained from Sarn Chemical Technology Co., Ltd. (Shanghai, China).

### 3.2. Synthesis of DPB

**Scheme 2:**
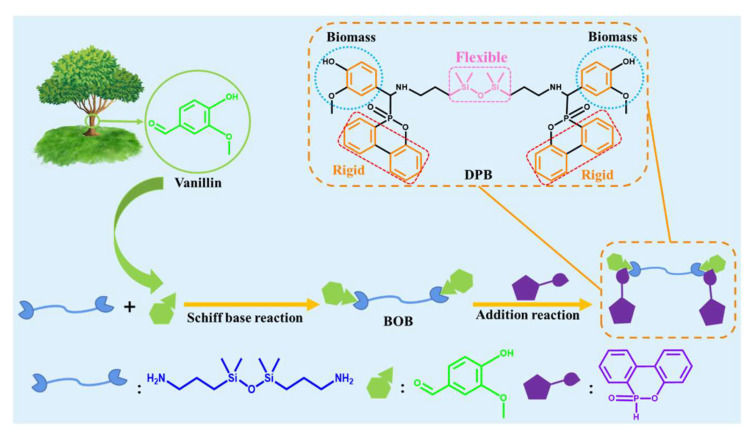
Diagram of the synthesis process of DPB.

First, 4.0 g of vanillin (26.3 mmol) and 30 mL of anhydrous ethanol were added to a three-necked flask. Then, 3.75 mL of BTDS (13.2 mmol) was dissolved in ethanol (10 mL) and slowly dropped into the three-necked flask. The reaction was stirred at 50 °C for 4 h. At the end of the reaction, most of the solvent was removed on a rotary evaporator at 60 °C, and then hexane was added and recrystallized. The lower layer was solvent-spun dry and dried under vacuum at 80 °C for 12 h to obtain the intermediate product (abbreviated as BOB). Then, 1.67 g of DOPO (7.8 mmol) was dissolved in ethanol (20 mL) and slowly added to the ethanol solution with 2 g of BOB (3.9 mmol), and the reaction was completed at 50 °C for 4 h. The crude product was dissolved in dichloroethane after rotary evaporation to remove ethanol and then washed 2–3 times with deionized water. After drying in a vacuum oven at 80 °C for 24 h, a novel biomass-containing intumescent flame retardant containing a rigid–flexible and multi-siloxane bridge structure (DPB) was obtained with a yield of 93.8%. ^1^H NMR (400 MHz, CDCl_3_, δ, ppm): δ = 7.28 (CDCl_3_), δ = 3.50 (H_2_O), δ = 0.08 (4H, H_1_), δ = 0.34 (2H, H_2_), δ = 1.22 (2H, H_3_), δ = 2.48 (2H, H_4_), δ = 3.74 (3H, H_8_), δ = 3.99 (1H, H_6_), δ = 4.24 (1H, H_5_), δ = 6.45–6.79 (3H, H_7_, H_11_, H_10_), δ = 6.79–8.05 (8H, H_12_), and δ = 9.81 (1H, H_9_). The synthesis steps are given in Scheme 2.

### 3.3. Preparation of DPB/EP Composite

Different proportions of DPB were added to 100 g of E-51 epoxy resin and stirred at 100 °C for 20 min to obtain a uniform mixture. The epoxy resin was then mixed with 25 g of DDM and stirred at 100 °C until the DDM was completely dissolved. Finally, the mixture was poured into a mold for segmental curing. The curing procedure was 2 h at 100 °C and 3 h at 150 °C. The formulations of DPB/EP containing different amounts of DPB are presented in Table 3.

### 3.4. Characterization

Fourier transform infrared (FTIR) spectra were attained using the Thermo Nicolet IS50 Spectrometer (Thermo Fisher Scientific, Waltham, MA, USA). The samples were tested using the ATR method, and the scan wavenumber range was 400–4000 cm^−1^. The number of scans was 32, and the resolution of the instrument was 0.09 cm^−1^.

The nuclear magnetic resonance (NMR) spectra were measured using a Bruker ARX 400 spectrometer (Bruker, Karlsruhe, Germany) by utilizing CDCl_3_ as a solvent.

The limiting oxygen index (LOI), the minimum percentage by volume of oxygen required by a specimen to maintain combustion equilibrium in a gas mixture of oxygen and nitrogen, was tested at room temperature on an HC-2C model tester (Jiangning Analytical Instrument Co., Ltd., Nanjing, China), and the specimen size was 130 × 6.5 × 3 mm^3^ using GB/T2406.2-2009 [69] as the standard. Five specimens were tested for each percentage, and the average value was taken.

Vertical burning (UL-94) was measured on a CZF-2 model tester (Jiangning Analytical Instrument Co., Ltd., Nanjing, China). The specimen size was 130 × 13 × 3 mm^3^ using GB/T2408-2008 [70] as the standard.

The combustion behavior was tested using an FTT0007 conical calorimeter (Fire Testing Technology, West Sussex, UK) according to ISO5660 [71]. The dimensions of specimens were 100 × 100 × 3 mm^3^, and the heat radiation power was 35 kW/m^2^.

The tensile and flexural tests were analyzed using a universal mechanical testing machine (SANS, Minneapolis, MN, USA) using GB/T 1040.2-2022 [72] and GB/T9341-2008 [73] as standards with a speed constant of 2 mm min^−1^. The specimen size for the dumbbell type in the tensile test was 100 × 10 × 3 mm^3^, and the specimen size for the rectangular type in the flexural test was 80 × 10 × 4 mm^3^.

The fracture surfaces of the samples and the char residue after cone calorimetric testing were scanned using a ZEISS Sigma 300 scanning electron microscope (SEM) (Carl Zeiss AG, Oberkochen, Germany), and the accelerating voltage was 3 kV.

Raman data were obtained using an Xplora-plus spectrometer (Horiba Jobin Yvon, Paris, France) using a 532 nm helium–neon laser line.

The thermal stability of DPB/EP with different stoichiometric ratios was analyzed by means of an SDTA851e thermogravimetric analyzer (TGA) (Mettler-Toledo, Zurich, Switzerland) under a N_2_ atmosphere with a heating rate of 10 °C/min.

The dielectric behavior of DPB/EP was measured using a Tonghui TH2838LCR tester (Tonghui Electronics, Changzhou, China). The test was conducted at room temperature at frequencies from 10^2^ Hz to 2 MHz.

X-ray photoelectron spectroscopy (XPS) was carried out on an ESCALAB 250XI electron spectrometer (Thermo Fisher Scientific, Waltham, MA, USA) to detect the bonding of the elements.

The elemental content was measured on a Vario EL Cube elemental analyzer (Elementar Analysensysteme GmbH, Frankfurt, Germany). A tin box was used for wrapping, and the combustion temperature was 1000 °C.

## 4. Conclusions

In this work, we synthesized a three-in-one biomass-containing intumescent flame retardant containing a rigid–flexible and multi-siloxane bridge structure (DPB) using siloxane as the gas source, DOPO as the acid source, and the biomass vanillin as the carbon source. DPB imparted excellent thermal stability and flame retardancy to the epoxy resin. When the content of DPB reached 7.5 phr, the pHRR and THR of DPB/EP decreased from 1184 kW/m^2^ and 155 MJ/m^2^ to 724 kW/m^2^ and 85.4 MJ/m^2^, respectively. The LOI value of DPB/EP was up to 30.9% and the UL-94 rating reached a V-0 rating when the DPB content was only 2.5 phr. This was because during the combustion process, the polyphosphoric acid generated by the decomposition could promote the dehydration and carbonization of the matrix, forming an expanded protective carbon layer, which acted as a physical barrier to prevent the transfer of volatiles and heat. The mechanical properties of DPB/EP were greatly improved by the combination of the rigid aromatic structure and the flexible Si-O bond in DPB. The tensile and flexural elongations at break of DPB/EP-7.5 were 77.2% and 105.3% higher than those of DPB/EP-0, respectively. More importantly, the dielectric constant of DPB/EP-7.5 was as low as 2.5–2.9, indicating that it was safer to use in electronic products. This was attributed to the fact that the Si-O bond in DPB effectively quenched the polarization behavior of the hydroxyl group, which effectively reduced the dielectric constant of DPB/EP. This paper proposed a practical solution for the preparation of epoxy resin composites with excellent flame retardancy, outstanding mechanical properties, and low dielectric constants, which will greatly broaden the application of EP in terms of electronic products.

## Figures and Tables

**Table 1:** TGA results of DPB/EP.

Sample	T_5%_ (°C)	T_max_ (°C)	RC_700_ (wt%)
DPB/EP-0	379 ± 1.3	394 ± 1.4	17.2 ± 0.2
DPB/EP-2.5	358 ± 1.5	385 ± 1.4	19.4 ± 0.3
DPB/EP-5	356 ± 2.0	381 ± 1.9	19.4 ± 0.3
DPB/EP-7.5	354 ± 1.8	378 ± 1.6	20.7 ± 0.2
DPB/EP-10	353 ± 1.7	379 ± 1.8	19.4 ± 0.1

**Table 2:** Summary of CCT data for DPB/EP.

Sample	TSP (m^2^)	THR (MJ/m^2^)	pHRR (kW/m^2^)	TTI (s)	MLR (g/s)
DPB/EP-0	26.1	155.5	1184	92	0.053
DPB/EP-2.5	25.6	103.3	1008	76	0.042
DPB/EP-5	25.0	101.1	984	77	0.040
DPB/EP-7.5	23.6	85.4	724	72	0.032
DPB/EP-10	24.7	93.8	884	69	0.035

**Table 3:** The formulation of DPB/EP.

Sample	DPB (g)	E-51 (g)	DDM (g)
DPB/EP-0	0	100	25.0
DPB/EP-2.5	2.5	100	25.0
DPB/EP-5	5	100	25.0
DPB/EP-7.5	7.5	100	25.0
DPB/EP-10	10	100	25.0

## Data Availability

Not applicable.
